# Regulatory Roles of PINK1-Parkin and AMPK in Ubiquitin-Dependent Skeletal Muscle Mitophagy

**DOI:** 10.3389/fphys.2020.608474

**Published:** 2020-12-03

**Authors:** Alex P. Seabright, Yu-Chiang Lai

**Affiliations:** ^1^School of Sport, Exercise and Rehabilitation Sciences, University of Birmingham, Birmingham, United Kingdom; ^2^Institute of Metabolism and Systems Research, University of Birmingham, Birmingham, United Kingdom; ^3^Mitochondrial Profiling Centre, University of Birmingham, Birmingham, United Kingdom; ^4^Medical Research Council (MRC) Versus Arthritis Centre for Musculoskeletal Ageing Research, University of Birmingham, Birmingham, United Kingdom

**Keywords:** mitophagy, mitochondrial fission, skeletal muscle, AMPK, ULK1, PINK1, Parkin, TBK1

## Abstract

The selective removal of damaged mitochondria, also known as mitophagy, is an important mechanism that regulates mitochondrial quality control. Evidence suggests that mitophagy is adversely affected in aged skeletal muscle, and this is thought to contribute toward the age-related decline of muscle health. While our knowledge of the molecular mechanisms that regulate mitophagy are derived mostly from work in non-muscle cells, whether these mechanisms are conferred in muscle under physiological conditions has not been thoroughly investigated. Recent findings from our laboratory and those of others have made several novel contributions to this field. Herein, we consolidate current literature, including our recent work, while evaluating how ubiquitin-dependent mitophagy is regulated both in muscle and non-muscle cells through the steps of mitochondrial fission, ubiquitylation, and autophagosomal engulfment. During ubiquitin-dependent mitophagy in non-muscle cells, mitochondrial depolarization activates PINK1-Parkin signaling to elicit mitochondrial ubiquitylation. TANK-binding kinase 1 (TBK1) then activates autophagy receptors, which in turn, tether ubiquitylated mitochondria to autophagosomes prior to lysosomal degradation. In skeletal muscle, evidence supporting the involvement of PINK1-Parkin signaling in mitophagy is lacking. Instead, 5′-AMP-activated protein kinase (AMPK) is emerging as a critical regulator. Mechanistically, AMPK activation promotes mitochondrial fission before enhancing autophagosomal engulfment of damaged mitochondria possibly via TBK1. While TBK1 may be a point of convergence between PINK1-Parkin and AMPK signaling in muscle, the critical question that remains is: whether mitochondrial ubiquitylation is required for mitophagy. In future, improving understanding of molecular processes that regulate mitophagy in muscle will help to develop novel strategies to promote healthy aging.

## Introduction

Skeletal muscle mitochondria are indispensable organelles that supply energy, in the form of ATP, to match metabolic and locomotive demands. To maintain optimal functioning in skeletal muscle, it is critical to remove damaged mitochondria through selective autophagy, known as mitophagy (Gan et al., 2018). While mitochondrial quality control is regulated by several mechanisms, including both mitochondrial biogenesis and mitophagy, evidence suggests that the latter is adversely affected during aging (Drake and Yan, 2017; Carter et al., 2018; Chen et al., 2018). At present, it is not possible to directly assess skeletal muscle mitophagy in humans. However, evidence suggests that skeletal muscle mitochondria accrue protein damage (Beltran Valls et al., 2015) and become functionally impaired (Trounce et al., 1989; Conley et al., 2000; Tonkonogi et al., 2003; Short et al., 2005) during human aging. The accumulation of damaged and dysfunctional mitochondria reported in these studies is consistent with the notion that mitochondrial clearance via mitophagy is compromised in aged muscle.

**FIGURE 1:**
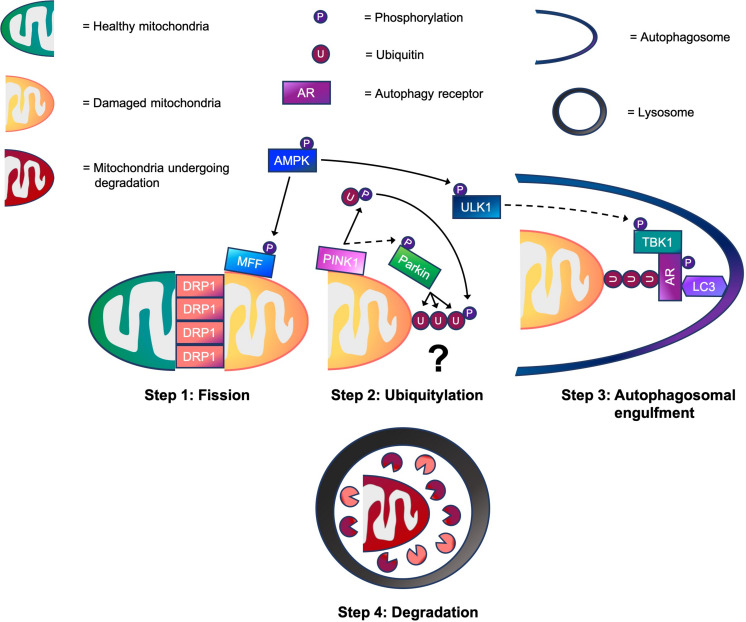
A working model of the four step mitophagy process in skeletal muscle. Step 1: Activation of 5’-AMP-activated protein kinase (AMPK) promotes mitochondrial fission via phosphorylation of MFF. This helps to recruit dynamin-related protein 1 (DRP1) to the outer mitochondrial membrane (OMM), enabling it to wrap around mitochondrial constriction sites to carry out scission, separating healthy and damaged mitochondria. Step 2: While the physiological conditions needed to activate PINK1-Parkin signaling for mitophagy in skeletal muscle are unknown, our data suggests that PINK1 accumulates on the OMM where it phosphorylates ubiquitin following CCCP-induced mitochondrial depolarization. This promotes the recruitment of Parkin E3 ubiquitin ligase to the OMM, which in turn, facilitates PINK1-mediated Parkin phosphorylation. Next, Parkin flags damaged mitochondria by ubiquitylating OMM proteins. (?) Despite our knowledge of these events, whether mitochondrial ubiquitylation is required for mitophagy in skeletal muscle is a critical question that warrants future investigation. Step 3: Meanwhile, AMPK also phosphorylates and activates ULK1. This enables the ULK1 complex to translocate to the mitochondria for its involvement in autophagosomal engulfment. ULK1 is suggested to phosphorylate and activate TANK-binding kinase 1 (TBK1). During autophagosomal engulfment, TBK1 activates autophagy receptors, such as optineurin (OPTN) and nuclear dot protein 52 (NDP52) that tether ubiquitylated mitochondria to autophagosomes. Step 4: Lastly, autophagosomes fuse with lysosomes for the degradation of damaged mitochondrial fragments. → = Signaling that occurs in skeletal muscle. ⇢ = Assumption based on signaling events in non-muscle cell lines.

Much of our knowledge regarding mitophagy and its molecular mechanisms is derived from work in immortalized, non-muscle cells lines. In these cells, it has become clear that ubiquitin-dependent, receptor-dependent, and cardiolipin-dependent mechanisms are capable of regulating mitophagy (Villa et al., 2018). Although signaling molecules such as, BNIP3, NIX/BNIP3L, FUNDC1, BCL2-L-13, and FKBP8 are implicated in receptor-dependent mitophagy, as is cardiolipin in cardiolipin-dependent mitophagy (Rodger et al., 2018), these are beyond the scope of this review. However, what is known about ubiquitin-dependent mitophagy in non-muscle cells is that it is comprised of four key steps: mitochondrial fission, ubiquitylation, autophagosomal engulfment, and degradation. Execution of these steps in sequence promotes efficient mitophagy (see Figure 1). Firstly, healthy and damaged mitochondria need to be separated via fission. Once separated, damaged mitochondria are marked with ubiquitin chains that act as a recognition signal for autophagy receptors. Autophagy receptors then tether ubiquitylated mitochondria to autophagic membranes, enabling autophagosomes to engulf damaged mitochondria. Finally, autophagosomes fuse with lysosomes for mitochondrial degradation.

From a mechanistic perspective, dynamin-related protein 1 (DRP1), mitochondrial fission factor (MFF), mitochondrial fission 1 protein (FIS1) and mitochondrial dynamics proteins 49/51 (MID49/51) have been shown to regulate the process of mitochondrial fission (Otera et al., 2010; Loson et al., 2013). Moreover, several E3 ubiquitin ligases, such as Parkin (Chan et al., 2011; Sarraf et al., 2013), MARCH5 (Chen et al., 2017), and MUL1 (Yun et al., 2014) are known to ubiquitylate outer mitochondrial membrane (OMM) proteins. Once mitochondria have been ubiquitylated they are recognized by autophagy receptors through their ubiquitin binding domain. There are five autophagy receptors named: next to BRCA1 gene 1 protein (NBR1) nuclear dot protein 52 (NDP52), optineurin (OPTN), sequestosome-1 (SQSTM1/p62), and tax1-binding protein 1 (TAX1BP1) recognize ubiquitylated mitochondria and facilitate autophagosomal engulfment prior to mitochondrial degradation (Yamano et al., 2016). Due to its high relevance in many human diseases (Bakula and Scheibye-Knudsen, 2020), multiple signaling pathways are proposed to regulate the aforementioned components in order to orchestrate the four step mitophagy process (Zimmermann and Reichert, 2017). However, in skeletal muscle, our understanding of mitophagy and the molecular mechanisms underlying each of its four steps has not been thoroughly explored.

Transmission electron microscopy (TEM) is the gold standard technique for detecting mitophagy occurrence because it enables direct visualization of mitochondria inside autophagic and lysosomal membranes. However, it is not widely used in skeletal muscle, mainly because operating TEM requires a high degree of technical expertise (Mcwilliams and Ganley, 2019). In recent years, a number of fluorescence-based mouse models [e.g., mito-QC (Mcwilliams et al., 2016), mt-Keima (Sun et al., 2015) and mitoTimer (Wilson et al., 2019)] have been developed to measure mitophagy *in vivo*. These ground-breaking innovations have enabled researchers to produce quantitative data in order to evaluate the level of mitophagy in skeletal muscle as well as in a range of other tissues and cell types. Alongside these, other techniques, such as mitophagy flux and mitotracker-lysotracker colocalization can also be used to assess mitophagy. These and other techniques have been well reviewed previously (Mcwilliams and Ganley, 2019). In the absence of such tools, the majority of research in skeletal muscle has assessed mitophagy signaling markers, such as Parkin and BCL2 interacting protein 3 (BNIP3) to indicate mitophagy (Schwalm et al., 2017; Brandt et al., 2018; Leermakers et al., 2018, 2019; Balan et al., 2019). However, by measuring these signaling markers alone, it is difficult to make reliable conclusions about mitophagy.

In order to advance understanding of mitophagy in skeletal muscle, we recently generated a stable C2C12 “mito-QC” cell line to study mitophagy (Seabright et al., 2020). This cell line also facilitates the assessment of mitochondrial morphology, including mitochondrial fission/fusion events. In terms of the underlying signaling mechanisms, the PINK1-Parkin signaling axis is known to be the classical pathway that regulates: mitochondrial fission, ubiquitylation, and autophagosomal engulfment during mitophagy. However, the vast majority of work investigating PINK1-Parkin signaling, has been conducted in cells that overexpress exogenous proteins to amplify ubiquitin signals (Narendra et al., 2008, 2010; Chan et al., 2011; Kane et al., 2014; Kazlauskaite et al., 2014; Heo et al., 2015, 2018; Richter et al., 2016). To circumvent this, we employed a tandem ubiquitin binding entity (TUBE) pull-down technique to enrich ubiquitin, allowing us to assess endogenous ubiquitylation status (Seabright et al., 2020). Interestingly, AMPK is also emerging as a key regulator of mitophagy via its involvement in mitochondrial fission and autophagosomal engulfment.

In this review we aim to integrate our recent findings into the current literature and evaluate how mitophagy is regulated in skeletal muscle through the steps of mitochondrial fission, ubiquitylation, and autophagosomal engulfment. Firstly, we will discuss the involvement of PINK1-Parkin signaling in mitochondrial fission, ubiquitylation, and autophagosomal engulfment. Afterward, we will explore the role of AMPK in mitochondrial fission and autophagosomal engulfment. Finally, we will highlight some of the important questions that are yet to be answered within the field.

## PINK1-Parkin-Mediated Mitophagy

The PTEN induced kinase 1 (PINK1)-Parkin signaling axis is considered to be the classical mitophagy pathway. This signaling pathway has been studied extensively in the context of neurodegeneration because mutations in human PINK1 (PARK6) (Valente et al., 2001, 2002) and Parkin (PARK2) (Kitada et al., 1998) are assumed to be causative for early onset Parkinson’s disease. In addition, abnormal mitophagy processing is thought to contribute toward the pathophysiology of Parkinson’s disease (Whitworth and Pallanck, 2017). Because of this, some studies have attempted to measure PINK1 or Parkin in skeletal muscle at the protein level to understand mitophagy (Borgia et al., 2017; Schwalm et al., 2017; Brandt et al., 2018; Carter et al., 2018; Leermakers et al., 2018, 2019; Balan et al., 2019; Drake et al., 2019). However, the abundance of a protein does not reflect its enzymatic activity. Furthermore, in models of aging (Leduc-Gaudet et al., 2019) and sepsis (Leduc-Gaudet et al., 2020), skeletal muscle Parkin overexpression has been shown to attenuate morphological and functional mitochondrial defects, possibly through enhanced mitophagy. These studies suggest that Parkin activation is sufficient to induce mitophagy in skeletal muscle. However, whether PINK1-Parkin signaling is crucial in the regulation of skeletal muscle mitophagy has not been thoroughly examined. In the next few sections, we will discuss the involvement of the PINK1-Parkin signaling pathway in mitophagy including the steps of mitochondrial fission, ubiquitylation and autophagosomal engulfment.

### PINK1-Parkin-Mediated Mitophagy in Non-muscle Cell Lines

What we have learnt from studies using non-muscle cell lines is that under basal conditions, PINK1 is continuously degraded through the N-end-rule pathway (Yamano and Youle, 2013). Conversely, insults such as: (a) the loss of the mitochondrial membrane potential (Narendra et al., 2008, 2010), (b) excessive production of reactive oxygen species (ROS) (Xiao et al., 2017a, b), and (c) mutations in mitochondrial DNA (mtDNA) (Suen et al., 2010), can activate PINK1 kinase activity. PINK1 then phosphorylates and activates Parkin’s ubiquitin E3 ligase activity, which labels damaged mitochondria with ubiquitin prior to autophagosomal engulfment and mitochondrial degradation. A detailed mechanistic summary of the PINK1-Parkin signaling pathway in mitophagy in non-muscle cell lines is provided elsewhere (Mcwilliams and Muqit, 2017; Pickles et al., 2018).

## PINK1-Parkin-Mediated Mitophagy in Skeletal Muscle

Only a few studies in the literature have evaluated the role of PINK1 and Parkin in skeletal muscle mitophagy. Borgia et al. (2017) reported that patients with spinal and bulbar muscular atrophy (SBMA) have increased PINK1 protein in mitochondria isolated from skeletal muscle (Borgia et al., 2017). Consistently, indirect measurements of mitophagy (co-localization of LC3 and ATPase) are also increased in SBMA patients, suggesting that the PINK1-mediated mitophagy may be activated under conditions of severe pathological stress in human skeletal muscle. Furthermore, Chen et al. (2018) reported that LC3-II flux in mitochondrial enriched skeletal muscle lysates is attenuated following endurance exercise in Parkin knock-out (KO) mice, suggesting that Parkin is required for exercise-induced mitophagy. However, because mitophagy was not directly measured in these studies, the reliability of the conclusions is limited. Interestingly, recent work from the Yan laboratory has shown that acute endurance exercise induces mitophagy in mice expressing the pMitoTimer reporter (Laker et al., 2017). However, using the same exercise protocol, the authors reported that PINK1 is not present in mitochondria enriched muscle lysates, suggesting that PINK1 is dispensable for mitophagy under these conditions (Drake et al., 2019). Thus, there is insufficient evidence to support the involvement of the PINK1-Parkin signaling pathway in skeletal muscle mitophagy. Notably, Mcwilliams et al. (2018) demonstrated that basal mitophagy occurs in muscle from both PINK1 KO and wild type mice on a mito-QC background, suggesting that under healthy, rested conditions, PINK1 is not required for mitophagy in the basal state. However, we cannot exclude the possibility that PINK1 may regulate disease-induced skeletal muscle mitophagy as different stimuli may induce different mechanisms of mitophagy.

### PINK1-Parkin Mediated Mitochondrial Fission

#### Non-muscle Cell Lines

Mitochondrial fission is a prerequisite step that separates damaged mitochondrial fragments for subsequent degradation (Toyama et al., 2016; Burman et al., 2017). Evidence suggests that both PINK1 and Parkin are able to regulate mitochondrial fission. In rat hippocampal neurons, overexpression of both PINK1 and Parkin induces fission, whereas knockdown of PINK1 has the opposite effect (Yu et al., 2011). Yang et al. (2008) have also shown that PINK1 knockdown increases the number of long tubular mitochondria, and this effect is suppressed by the overexpression of DRP1 or FIS1. In another study using COS-7 cells, overexpression of an inactive DRP1 variant (DRP1 K38A) significantly attenuated the pro-fission effects of individual PINK1 and Parkin overexpression (Buhlman et al., 2014). Collectively, these data suggest that PINK1 and Parkin regulate DRP1-mediated mitochondrial fission.

#### Skeletal Muscle

Recent work conducted by Favaro et al. (2019) revealed that mitochondrial volume is not significantly altered in DRP1-deficient muscle, but the average size of individual organelles is greater. These data indicate that DRP1 deletion induces fusion of the mitochondrial network, resulting in larger, but fewer mitochondria. By contrast, optic atrophy protein 1 (OPA1) regulates fusion of the inner mitochondrial membrane. Interestingly, deletion of OPA1 in skeletal muscle results in smaller mitochondria with dilated cristae (Tezze et al., 2017) whereas mitochondria are larger and elongated following inhibition of DRP1 when OPA1 is absent (Romanello et al., 2019). Taken together, these findings suggest that inhibition of mitochondrial fission is dominant over the inhibition of mitochondrial fusion.

While the signaling events that precede DRP1/OPA1-mediated mitochondrial fission/fusion in skeletal muscle remain largely unexplored, Parkin’s role in these processes has been investigated in a few studies. Recently, Gouspillou et al. (2018) demonstrated that Parkin deletion increases DRP1 protein content in skeletal muscle while reducing levels of the pro-fusion protein MFN2. However, in another study, loss of Parkin was shown to attenuate DRP1 and FIS1 protein content without altering pro-fusion proteins (Peker et al., 2018). While the changes reported in these studies highlight possible interplay between Parkin and mitochondrial fission/fusion, whether activation of PINK1-Parkin signaling induces mitochondrial fragmentation in skeletal muscle requires further investigation.

### PINK1-Parkin Mediated Mitochondrial Ubiquitylation

#### Non-muscle Cell Lines

Labeling damaged mitochondria with ubiquitin molecules is an important step that helps to recruit autophagy receptors for autophagosomal engulfment (Yamano et al., 2016). The reason why PINK1-Parkin signaling is a critical mediator of mitophagy is because activation of Parkin’s ubiquitin E3 ligase activity leads to mitochondrial ubiquitylation (Lee et al., 2010). In response to mitochondrial depolarization, PINK1 is stabilized and activated on the outer mitochondrial membrane (OMM). PINK1 then phosphorylates ubiquitin (Kane et al., 2014; Kazlauskaite et al., 2014; Koyano et al., 2014), which in turn, recruits Parkin to the OMM (Narendra et al., 2010). Furthermore, binding of phospho-ubiquitin to Parkin (Wauer et al., 2015) along with PINK1-mediated Parkin phosphorylation, maximally activates Parkin E3 ligase activity (Kondapalli et al., 2012; Kazlauskaite et al., 2015). This enables Parkin to label damaged mitochondrial by ubiquitylating OMM proteins, such as: CDGSH iron sulfur domain 1 (CISD1); mitofusin-1/2 (MFN-1/2); translocase of outer membrane 70 kDa subunit (TOM70); mitochondrial fission 1 protein (FIS1); and, voltage-dependent anion channel 1 (VDAC1) (Chan et al., 2011; Sarraf et al., 2013).

#### Skeletal Muscle

While the PINK1-Parkin signaling pathway is the most widely studied mechanism of mitochondrial ubiquitylation, the necessity of this pathway for mitophagy in skeletal muscle is currently undefined. This is primarily due to the lack of appropriate tools to study the activities of PINK1 kinase and Parkin E3 ligase. The main reason why there are so few studies investigating the role of PINK1 in skeletal muscle mitophagy is because many of the commercially available antibodies are insensitive or unspecific when detecting endogenous PINK1 (Walsh et al., 2018). Furthermore, because Parkin is reported to translocate to mitochondria for both mitophagy (Narendra et al., 2008) and mitochondrial biogenesis (Kuroda et al., 2006; Stevens et al., 2015; Leduc-Gaudet et al., 2019) simply measuring mitochondrial Parkin content does not determine which process Parkin is involved in. Thus, developing tools to assess the activities of PINK1 kinase and Parkin E3 ligase will help to improve our understanding of the role of PINK1-Parkin signaling in skeletal muscle mitophagy.

There are many advantages to using ubiquitin binding domain (UBD) pull-down assays, such as TUBE to study endogenous ubiquitylation events including those associated with PINK1 kinase and Parkin E3 ligase (Emmerich and Cohen, 2015). TUBE has an extremely high affinity and can capture all mono and poly-ubiquitin chain types including ubiquitylated proteins in both cell and tissue lysates (Emmerich and Cohen, 2015). This enrichment step is important because it also captures proteins that are only ubiquitylated to a low stoichiometry. Furthermore, TUBE has been shown to protect poly-ubiquitin chains and ubiquitylated proteins from a range of deubiquitylases (DUBs) (Hjerpe et al., 2009) while circumventing antibody heavy and light chains, which can interfere with western blotting analysis (Emmerich and Cohen, 2015). Lastly, by combining TUBE with a HaloTag system for example, it is possible to perform extensive washing after ubiquitin enrichment in order to minimize non-specific binding (Emmerich and Cohen, 2015). Other methods, such as proteomics coupled with ubiquitin remnant motif antibodies (Cell Signaling Technology), can also be used to assess ubiquitylation events in skeletal muscle as described by Parker et al. (2020).

We recently implemented a tandem ubiquitin binding entity (TUBE) pulldown technique with a HaloTag system to enrich ubiquitin and simultaneously capture all the ubiquitylated proteins in C2C12 skeletal muscle myotubes (Seabright et al., 2020). Following SDS-PAGE and protein transfer, we then used antibodies specific to phosphorylated ubiquitin at Ser 65 and CISD1 to measure intracellular PINK1 kinase activity and Parkin E3 ligase activity, respectively (Seabright et al., 2020). In this experiment, we found that endogenous activities of PINK1 kinase and Parkin E3 ligase were increased 6, 12, and 24 h following CCCP-induced mitochondrial depolarization in C2C12 myotubes (Seabright et al., 2020). However, because prolonged (≥6 h) CCCP treatment is not physiological, these findings also indicate that activation of PINK1-Parkin signaling may not occur under physiological conditions *in vivo*.

So far, no study has been able to clearly demonstrate that PINK1-Parkin-mediated mitochondrial ubiquitylation occurs in skeletal muscle. Since mitochondrial ubiquitylation is a critical step for the recognition of damaged mitochondria in the lead up to their degradation, we suggest that TUBE ubiquitin enrichment techniques be used to address this question.

### PINK1-Parkin-Mediated Autophagosomal Engulfment

#### Non-muscle Cell Lines

Activation Parkin E3 ligase activity labels damaged mitochondria with ubiquitin, helping to recruit autophagy receptors for autophagosomal engulfment. The formation of poly-ubiquitin chains on damaged mitochondria leads to the recruitment of autophagy receptors, including NBR1, NDP52, OPTN, SQSTM1/p62, and TAX1BP1 (Yamano et al., 2016). Because these autophagy receptors possess a ubiquitin-binding domain as well as a LC3-interacting region (LIR) motif, they have the capacity to link ubiquitylated mitochondria to autophagosomal LC3. Mechanistically, NDP52 and OPTN are the essential receptors for mitophagy with some contribution from TAX1BP1, whereas NBR1 and p62 are dispensable (Heo et al., 2015; Lazarou et al., 2015). The binding capacity of OPTN with poly-ubiquitin chains is enhanced by TBK1 which phosphorylates OPTN at multiple serine residues (Richter et al., 2016). In the other study, Vargas et al. (2019) have also shown that TBK1 activation promotes NDP52-mediated recruitment of the ULK1 complex during autophagosomal engulfment. Importantly, studies have also shown that TBK1 activation following its phosphorylation at Ser 172 (Larabi et al., 2013) requires the activation of PINK1-Parkin signaling, which in turn, generates poly-ubiquitin chains on depolarized mitochondria (Heo et al., 2015; Lazarou et al., 2015). Based on these reports, it is clear that TBK1 plays a critical role in autophagosomal engulfment of damaged mitochondria via its association with OPTN and NDP52.

#### Skeletal Muscle

In skeletal muscle, however, recent work from our laboratory suggests that TBK1 phosphorylation and activation is independent of both PINK1 and Parkin activation (Seabright et al., 2020). Using a time course experiment, we observed that TBK1 phosphorylation occurs prior to the activation of PINK1 kinase and Parkin E3 ligase activity. We also demonstrated that TBK1 phosphorylation occurs normally in PINK1 knockout HeLa cells following CCCP treatment. We suspect that the disparity between our data and findings generated in non-muscle cell lines may be due to the use of overexpression systems employed in other studies. HeLa cells lack native Parkin expression (Denison et al., 2003). Stably expressing non-native Parkin, has been shown to induce artificial activation of its E3 ligase activity, particularly when fused with exogenous tags at its N-terminus (Burchell et al., 2012). Thus, we believe that in skeletal muscle TBK1 is acutely activated following mitochondrial depolarization in a PINK1-Parkin independent manner. However, this conclusion also raises the question as to which other E3 ligases, excluding Parkin, elicit mitochondrial ubiquitylation to promote autophagy receptor recognition and interaction with autophagic membranes.

### A Summary of the PINK1-Parkin Signaling Pathway in Skeletal Muscle Mitophagy

So far, evidence supporting the involvement of PINK1-Parkin signaling in the molecular regulation of skeletal muscle mitophagy is limited. This is mainly due to lack of tools to study endogenous PINK1-Parkin signaling. The integration of new techniques that enrich ubiquitin, including TUBE, will help to provide novel insight into PINK1-Parkin signaling in skeletal muscle. As evidence for this, prolonged CCCP treatment leads to the activation of PINK1-Parkin signaling in C2C12 skeletal muscle myotubes (Seabright et al., 2020). Although our work indicates that the PINK1-Parkin signaling axis is functionally active in skeletal muscle, CCCP treatment is not physiological. Therefore, employing techniques to enrich ubiquitin in future studies, may help to identify physiological conditions that activate PINK1-Parkin signaling as well as determining whether mitochondrial ubiquitylation in skeletal muscle is required for mitophagy.

## AMPK-Mediated Mitophagy

### AMPK in Skeletal Muscle

5′-AMP-activated protein kinase (AMPK) is a master energy-sensing kinase that becomes activated in response to rising AMP levels following ATP hydrolysis. Under these conditions, AMPK activates glucose and fatty acid utilization (Garcia and Shaw, 2017) while inhibiting pro-growth signals (Inoki et al., 2003) in order to replenish ATP levels. Mitochondria act as the power plant in every cell and work to maintain ATP homeostasis. Therefore, it is not surprising that a strong link exists between AMPK signaling and mitochondrial biology. AMPK activation has been shown to promote mitochondrial biogenesis via the phosphorylation and activation of peroxisome proliferator-activated receptor gamma coactivator 1-alpha (PGC-1α) (Jäger et al., 2007). In recent years, AMPK has also been suggested to regulate mitochondrial fission (Toyama et al., 2016) and coordinate mitophagy (Laker et al., 2017) to help maintain pools of high functioning mitochondria.

### AMPK-Mediated Mitophagy in Non-muscle Cell Lines

Egan et al. (2011) first revealed that AMPK is involved in mitophagy. In their study, not only did cells expressing non-phosphorylatable ULK1 mutants contain more mitochondria, but a higher proportion of these mitochondria were enlarged with altered cristae. Others have subsequently shown that AMPK-mediated phosphorylation of ULK1 at Ser 555 regulates its activation and translocation to mitochondria for its involvement in mitophagy (Egan et al., 2011; Tian et al., 2015). These early studies indicate that AMPK-mediated phosphorylation of ULK1 facilitates mitophagy.

### AMPK-Mediated Mitophagy in Skeletal Muscle

Laker et al. (2017) were the first to demonstrate that AMPK is involved in skeletal muscle mitophagy using mice expressing a muscle-specific dominant-negative form of the catalytic α2 subunit of AMPK (dnTG). Interestingly, mitophagy was shown to be transiently elevated 6 h after a bout of treadmill running in wild type, but not dnTG, mice (Laker et al., 2017). Downstream of AMPK, the authors also showed that ULK1 is required for exercise-induced mitophagy (Laker et al., 2017). To advance these findings further, we recently demonstrated that specific, pharmacological activation of AMPK induces mitophagy in C2C12 skeletal muscle myoblasts stably expressing the mito-QC reporter (Seabright et al., 2020). As mentioned previously, basal mitophagy occurs when AMPK is suggested to be inactive under resting conditions. This indicates that other currently unknown signaling pathways are capable of regulating mitophagy in response to different stimuli.

### AMPK Mediated Mitochondrial Fission

#### Non-muscle Cell Lines

The process of mitochondrial fission is a critical step during mitophagy as it helps to separate healthy and damaged mitochondrial fragments. AMPK’s involvement in mitochondrial fission was first suggested by the Sakamoto laboratory, who used a proteomic approach to reveal that mitochondrial fission factor (MFF) is an AMPK substrate (Ducommun et al., 2015). Later, Toyama et al. (2016) used AMPK α1/α2 double knockout U2OS cells with specific (A769662 and AICAR) AMPK activators to demonstrate that AMPK activation induces mitochondrial fission. Along with mitochondrial fission protein 1 (FIS1) and mitochondrial dynamics proteins 49/51 (MID49/51), MFF functions as a key receptor for the dynamin-related protein 1 (DRP1) on the outer mitochondrial membrane (OMM) (Otera et al., 2010; Loson et al., 2013). Following its activation and translocation to the mitochondria, DRP1 wraps around mitochondrial constriction sites to enable scission. Interestingly, Toyama et al. (2016) also showed that AMPK-mediated phosphorylation of MFF at Ser 155 and 172 is required for DRP1 recruitment to the OMM. These findings indicate that AMPK-mediated phosphorylation of MFF induces DRP1 translocation to mitochondria to facilitate mitochondrial scission.

#### Skeletal Muscle

In agreement with observations made in non-muscle cell lines mentioned above, we have shown that AMPK activation by 991 induces phosphorylation of MFF and promotes the accumulation of smaller, fragmented mitochondria, indicating fission (Seabright et al., 2020). To the best of our knowledge this is the first study showing that MFF phosphorylation in C2C12 skeletal muscle myoblasts is AMPK dependent. Furthermore, although phosphorylation of DRP1 at Ser 616 is known to promote DRP1-mediated mitochondrial scission (Taguchi et al., 2007), Laker et al. (2017) have shown that AMPK is not responsible for exercise-induced phosphorylation of DRP1 at this site. These data suggest that assessing the phosphorylation status of both MFF and DRP1 can be used as a marker to indicate mitochondrial fission. While phosphorylation of MFF has been shown to mediate the recruitment of DRP1 to the OMM in non-muscle cell lines (Otera et al., 2010; Loson et al., 2013), this phenomenon has not been verified in skeletal muscle.

### AMPK and Mitochondrial Ubiquitylation

#### Non-muscle Cell Lines

As mentioned previously, labeling of damaged mitochondria with ubiquitin is essential to recruit autophagy receptors for autophagosomal engulfment. However, how AMPK is involved in mitochondrial ubiquitylation is still unclear. To us, it is surprising that AMPK reportedly activates both PINK1 kinase and Parkin E3 ligase activity through phosphorylation. Wang et al. (2018) showed that AMPKα2 phosphorylates PINK1 at Ser 495. This AMPK-mediated PINK1 phosphorylation induces its kinase activity, which leads to increased Parkin phosphorylation at Ser 65 in cardiomyocytes (Wang et al., 2018). In the other study, Lee et al. (2019) showed that AMPK can also phosphorylate Parkin Ser 9 to induce Parkin’s ubiquitin E3 ligase activity toward its substrate, RIPK3. These studies suggest possible interplay between AMPK and PINK1-Parkin signaling. However, evidence demonstrating that AMPK activation leads to mitochondrial ubiquitylation and/or Parkin-mediated OMM protein ubiquitylation is lacking.

#### Skeletal Muscle

By taking advantage of TUBE ubiquitin enrichment, we recently demonstrated that the intracellular activities of PINK1 kinase and Parkin E3 ligase are not increased following AMPK activator treatment in C2C12 skeletal muscle myotubes, despite significant induction of mitophagy (Seabright et al., 2020). We also showed no detectable mitochondrial depolarization under these conditions, suggesting that PINK1-Parkin signaling is not activated (Seabright et al., 2020). Although our data suggests that PINK1-Parkin signaling is not required for AMPK-mediated mitophagy, we have not yet tested whether mitochondrial ubiquitylation occurs under these conditions. Given that all five of the aforementioned autophagy receptors that facilitate autophagosomal engulfment have ubiquitin binding domains (Yamano et al., 2016), determining whether mitochondrial ubiquitylation is required for autophagy receptor recognition is the next critical question that should be addressed. If it is confirmed that mitochondrial ubiquitylation is required for mitophagy, it is important to determine which E3 ubiquitin ligases, other than Parkin, are responsible for this process.

### AMPK and Autophagosomal Engulfment

#### Non-muscle Tissue

OPTN and NDP52 are the principle autophagy receptors that tether ubiquitylated mitochondria to autophagic membranes via their ubiquitin binding and LC3 interacting domains, respectively (Heo et al., 2015; Lazarou et al., 2015). As previously mentioned, TBK1 activation is required to facilitate autophagosomal engulfment of ubiquitylated mitochondria by activating OPTN and NDP52. Interestingly, a recent study suggests that AMPK is able to activate TBK1 via ULK1 in adipose tissue (Zhao et al., 2018). However, in this study, AMPK-mediated phosphorylation and activation TBK1 was not investigated in the context of mitophagy.

#### Skeletal Muscle

While investigating the signaling mechanisms underlying skeletal muscle mitophagy in C2C12 myotubes, we observed similar phosphorylation patterns of both AMPK and TBK1 in response to indirect and specific AMPK activation (Seabright et al., 2020). To validate the cause-effect relationship between AMPK and TBK1, we used CRISPR-Cas9 generated AMPK α1/α2 knockout cells to demonstrate that TBK1 phosphorylation is AMPK dependent (Seabright et al., 2020). These data suggest that activation of AMPK promotes autophagosomal engulfment via TBK1 activation. Moreover, the findings of Zhao et al. (2018) further suggest that AMPK activates TBK1 via ULK1-mediated phosphorylation. Although these data suggest that AMPK-ULK1-TBK1 signaling occurs in skeletal muscle cells, the functional relevance of TBK1 in autophagosomal engulfment of damaged mitochondria is yet to be determined.

### A Summary of AMPK-Mediated Mitophagy in Skeletal Muscle

AMPK is emerging as a major regulator of mitophagy in skeletal muscle. Mechanistically, AMPK activation promotes mitochondrial fission before enhancing autophagosomal engulfment of damaged mitochondria possibly via TBK1. Further research is needed to verify the functional relevance AMPK-mediated TBK1 activation in autophagosomal engulfment of damaged mitochondria and therefore skeletal muscle mitophagy.

## A Working Model of Mitophagy in Skeletal Muscle

In this review, we summarize the molecular mechanisms that regulate skeletal muscle mitophagy in four key steps: mitochondrial fission, ubiquitylation, autophagosomal engulfment, and degradation (see Figure 1). We reveal that AMPK plays a vital role in the coordination of mitophagy and controls molecular mechanisms associated with mitochondrial fission via MFF and autophagosomal engulfment via ULK1 and TBK1. Although Parkin has been shown to induce mitochondrial ubiquitylation in skeletal muscle (Chen et al., 2018), there are no studies demonstrating that PINK1 is responsible for Parkin’s activation. Thus, such evidence supporting the activation of PINK1-Parkin signaling in mitochondrial ubiquitylation during mitophagy in skeletal muscle under physiological conditions is currently lacking. While TBK1 may be a point of convergence between AMPK and PINK1-Parkin signaling during mitophagy, the critical question that remains is whether mitochondrial ubiquitylation is required for mitophagy in muscle. Gaining a deeper understanding of molecular mechanisms that control mitophagy in skeletal muscle will help to identify important signaling molecules that regulate this process. Such knowledge can then be used to help develop novel therapies, including pharmacological strategies, to combat defective mitophagy in muscle and thus promote healthy aging.

## Future Directions

As highlighted throughout this review, crucial questions remain unanswered within this field are: (1) whether the step of mitochondrial ubiquitylation is required for mitophagy in skeletal muscle, and (2) under what physiological conditions is PINK1-Parkin signaling is activated for mitophagy. To help address the question outlined above, we recommend employing the TUBE ubiquitin enrichment technique described in this review to help facilitate the assessment of mitochondrial ubiquitylation, as well as PINK1 kinase and Parkin E3 ligase activities. Furthermore, using a newly developed fluorescence-based mouse model, such as mito-QC (Mcwilliams et al., 2016) will enable quantifiable read-outs of lysosome-dependent mitochondrial degradation to be made. In parallel, it would also be interesting to study mitochondrial fission and autophagosomal engulfment alongside their respective signaling markers (e.g., MFF, DRP1, ULK1, and TBK1) that are highlighted in this review.

## Author Contributions

APS: conceptualization, visualization, writing original draft, writing review, and editing. Y-CL: conceptualization, writing review, and editing. Both authors contributed to the article and approved the submitted version.

## Conflict of Interest

The authors declare that the research was conducted in the absence of any commercial or financial relationships that could be construed as a potential conflict of interest.

## Abbreviations

AMP: adenosine monophosphate
AMPK: 5’-AMP-activated protein kinase
ATP: adenosine triphosphate
BNIP3: BCL2/adenovirus E1B 19 kDa protein-interacting protein 3
CCCP: carbonyl cyanide m-chlorophenyl hydrazine
CISD1: CDGSH iron sulfur domain 1
DRP1: dynamin-related protein 1
FIS1: mitochondrial fission protein 1
FKBP8: Peptidyl-prolyl cis-trans isomerase FKBP8
FUNDC1: FUN14 domain-containing protein 1
HEK293: human embryonic kidney 293 cells
HeLa: Henrietta Lacks cells
LC3: microtubule-associated protein 1A/1B-light chain 3
LIR: LC3-interacting region
MFF: mitochondrial fission factor
MFN-1/2: mitofusin-1/2
MID49/51: mitochondrial dynamics proteins 49/51
NBR1: next to BRCA1 gene 1 protein
NDP52: nuclear dot protein 52
NIX/BNIP3L: BCL2/adenovirus E1B 19 kDa protein-interacting protein 3-like
OPA1: optic atrophy protein 1
OMM: outer mitochondrial membrane
OPTN: optineurin
PGC-1 α: peroxisome proliferator-activated receptor gamma coactivator 1-alpha
PINK1: PTEN-induced kinase 1
RIPK3: RIP-like protein kinase 3
SBMA: spinal and bulbar muscular atrophy
Ser: serine
SQSTM1/p62: sequestosome-1
TAX1BP1: tax1-binding protein 1
TBK1: TANK-binding kinase 1
TEM: transmission electron microscopy
TOM70: translocase of outer membrane 70 kDa subunit
TUBE: tandem ubiquitin binding entity
ULK1: unc-51 like autophagy activating kinase 1
U2OS: human bone osteosarcoma epithelial cells
VDAC1: voltage-dependent anion channel 1.
